# Use of systemic hormonal contraception and risk of depression: a registry-based study from Finland

**DOI:** 10.1007/s10654-025-01267-0

**Published:** 2025-07-02

**Authors:** Elena Toffol, Timo Partonen, Oskari Heikinheimo, Anna But, Antti Latvala, Jari Haukka

**Affiliations:** 1 Department of Public Health, Clinicum, Faculty of Medicine, University of Helsinki, PO BOX 20, Helsinki, 00014 Finland; 2 Department of Healthcare and Social Welfare, Finnish Institute for Health and Welfare, Helsinki, Finland; 3 Department of Obstetrics and Gynaecology, University of Helsinki, Helsinki University Hospital, Helsinki, Finland; 4 Institute of Criminology and Legal Policy, University of Helsinki, Helsinki, Finland

**Keywords:** Women, Girls, Adolescents, Contraceptive, Fertile-age, Combined hormonal contraceptives

## Abstract

Earlier findings on the relationship between use of hormonal contraception (HC) and depressive symptoms and disorders are contradictory. Thus, we assessed the associations of use of different types of systemic hormonal contraceptives in the six preceding months with the risk of depression in women aged 15–49 years. Data were obtained from national registers in Finland. All cases of depression in the years 2018–2019 were identified in a population-based cohort of women. We used a nested case-control design with 1:4 ratio (*n* = 117,360 cases) and applied multivariable conditional logistic regression models. During the follow-up a total of 23,480 new cases with the diagnosis of depression were observed (incidence rate: 21.7, 95% confidence interval = 21.5–22.0 per 1000 person-years). Use of HC in the six preceding months, specifically that of combined hormonal contraceptives (containing gestodene and ethinylestradiol, drospirenone and ethinylestradiol, and nomegestrol and estradiol), was significantly associated with a lower risk of depression compared to non-use when controlling for marital status, socioeconomic status, education, recent delivery, recent psychiatric hospitalization, chronic diseases, use of psychiatric medications (excluding antidepressants) and former use of HC (odds ratio: 0.90, 95% confidence interval = 0.85–0.95; 0.86, 95% confidence interval = 0.81–0.91, respectively). Current use of progestogen-only preparations (norethisterone, levonorgestrel, desogestrel) was not associated with depression. This pattern was evident in all age groups, including adolescent girls. HC use appeared not associated with an increased risk of depression in fertile-aged women and across all age groups, including adolescent girls.

**Supplementary Information:**

The online version contains supplementary material available at 10.1007/s10654-025-01267-0.

## Introduction

More than 970 million fertile-aged women use contraception globally [1], and approximately a third of them use a hormonal method [2]. Albeit its contraceptive and health benefits [3], use of hormonal contraception (HC) has been linked to adverse effects. In particular, the relationship between the use of HC and mood symptoms, such as mood changes and onset of depressive symptoms and disorders, has been largely debated. Mood changes or symptoms associated with HC use are common, and are a common cause of dissatisfaction, irregular use, and discontinuation of contraception [4]. However, not all women experience mood symptoms while on HC. Rather, it is likely that individual vulnerability, possibly related to a pre-existing mental ill-health or sensitivity to hormonal fluctuations and/or other reproductive events [5–12], makes a subgroup of women more likely to experience mood symptoms while on HC. Moreover, given the rapid development of new hormonal preparations in different combinations, doses and routes of administration, women exposed to different types of HC are likely to experience different profiles of adverse effects.

Recently, results of large observational studies conducted in the Nordic countries and based on data of approximately one million women followed up longitudinally for 1–14 years, indicated that use of HC (containing ethinylestradiol in combination with levonorgestrel, desogestrel, gestodene, drospirenone or cyproterone acetate, natural estrogen in combination with dienogest, as well as progestin only products) was associated with an increased risk of developing depressive disorders or using psychotropic medications (antidepressants or anxiolytics, sedatives, and hypnotics). The most pronounced associations were seen among adolescents [13–15]. A large population-based study conducted in over 250,000 women from the UK biobank reported similar findings, although lacking information on different HC types [16]. Moreover, a study of over 1200 women in the USA suggested that those who had started their oral contraceptive (OC) use (with no distinction of different products) in adolescence were more likely to develop depression in adulthood [17]. However, other studies with similar or different designs did not support such findings, or came to opposite conclusions [15, 18–21]. For example, a Swedish register-based cohort study found lower or no increased risk of depression among women using combined oral contraceptives (COCs) and progestogen-only pills (POP) in adults, but increased risk among adolescents using POPs, contraceptive patch/vaginal ring, implant or a levonorgestrel intrauterine device [15]. However, most of the studies to date failed to include information on specific types of hormonal preparations and doses, and, when available, conflicting results have been obtained [22].

Thus, the aim of our study was to examine the risk of developing depressive disorders and/or symptoms in relation to current use of different types of HC in a large cohort inclusive of all fertile-aged women using contraception in Finland, and a same-size matched reference cohort of non-users, being followed-up for two years.

## Materials and methods

### Study population and design

This study was conducted in connection with a larger register-based study of HC use in Finland [23]. The original population, selected on the basis of the unique personal identification number given at birth or at immigration to each person permanently residing in Finland, included all women aged 15–49 years who redeemed at least one HC prescription (Anatomical Therapeutic Chemical -ATC- codes: G02B, “contraceptives for topical use”; G03A, “hormonal contraceptives for systemic use”; G03HB, “antiandrogens and estrogens”) in 2017 (*n* = 294,445), as recorded in the Prescription Centre in the Kanta Services. In addition, we selected, in a 1:1 ratio, a reference group of women of same age and municipality of residence, with no redeemed HC prescriptions in 2017 (HC non-users). Women (*n* = 89) who had any redeemed prescription for emergency contraception (ATC code “G03AD”, usually available without prescription in Finland) and their matched reference individuals were excluded, leaving a final population of 588,712 women, corresponding to approximately half of the female population of that age group living in Finland. The study was reviewed by the Ethics Committee of the Faculty of Medicine, University of Helsinki (3/2018). Because this is a register-based study, no individual consent is needed.

We followed the HC use of all these women until the end of 2019, with a maximum length of follow-up of two years through the Prescription Centre. The primary endpoint events were defined as a first hospitalization or visit to specialized outpatient care (as recorded in the Care Register for Health Care) due to depressive disorder (International Classification of Diseases, Tenth Revision, ICD-10 diagnosis “F32”–“Depressive episode”- or “F33”– “Recurrent depressive disorder”-) or a primary health-care contact due to depression (as recorded in the Register of Primary Health Care visits; ICD-10 diagnosis “F32” or “F33”, or International Classification of Primary Care-2nd Edition, ICPC-2, “P76” -“Depressive disorder”-). Death, emigration from Finland or end of follow-up were defined as censoring events. Prevalent cases (with an endpoint event in 2016 or 2017, i.e., before the start of follow-up) were excluded from the study (*n* = 35,102).

The remaining population was used as a sampling frame for identifying all incident cases of depression in 2018–2019, and thus building a nested 1:4 case-control study, matched on birth year, aimed to explore the risk of depression related to current (i.e., in the six months before the event) HC use.

## Register data and variables

From Finnish national registers we obtained information on age, municipality of residence, civil status, socioeconomic status and highest level of education on 31 December 2017 (Statistics Finland); recent deliveries within the previous two years (Medical Birth Register); a cancer diagnosis in the previous five years (Finnish Cancer Registry); and special reimbursement rights for chronic diseases (diabetes, multiple sclerosis, epilepsy, severe psychiatric disorders, connective tissue diseases, ulcerative colitis or Crohn’s disease) from the Social Insurance Institution of Finland (Kela). From the Prescription Centre we obtained information on redeemed HC prescriptions in 2017, on HC use in the period 2018–2019, and on use of psychotropic medications in the period 2016–2020 (ATC codes: N05A, antipsychotics; N05B, anxiolytics; N05C, hypnotics and sedatives; N06A, antidepressants; N06B, psychostimulants; N06C, psycholeptics and psychoanaleptics in combination). Only ATC codes with at least five individuals in all categories were used in statistical analyses. Use of each substance (hormonal contraceptives and psychotropic drugs) was defined as two or more redeemed prescriptions in a 180-day period. Additionally, to exclude a bias related to women stopping HC because of early onset side effects, sensitivity analyses were conducted with HC use defined as one or more redeemed prescriptions in a 180-day period.

In the nested case-control design, for each HC substance we defined a categorical variable as follows: non-user (no use in the 180 days before the depression event) and current user (use in 1–180 days before the event). The HC methods of interest and available in Finland in 2018 are summarized in Table S1. Contraceptive implants and the levonorgestrel-releasing intrauterine system were excluded, because they can be used for up to three or five years and are often provided free-of-charge by individual communities, thus not necessitating individual prescription (and as such they are not completely covered in the Prescription Centre database).

## Statistical analyses

**Fig. 1:**
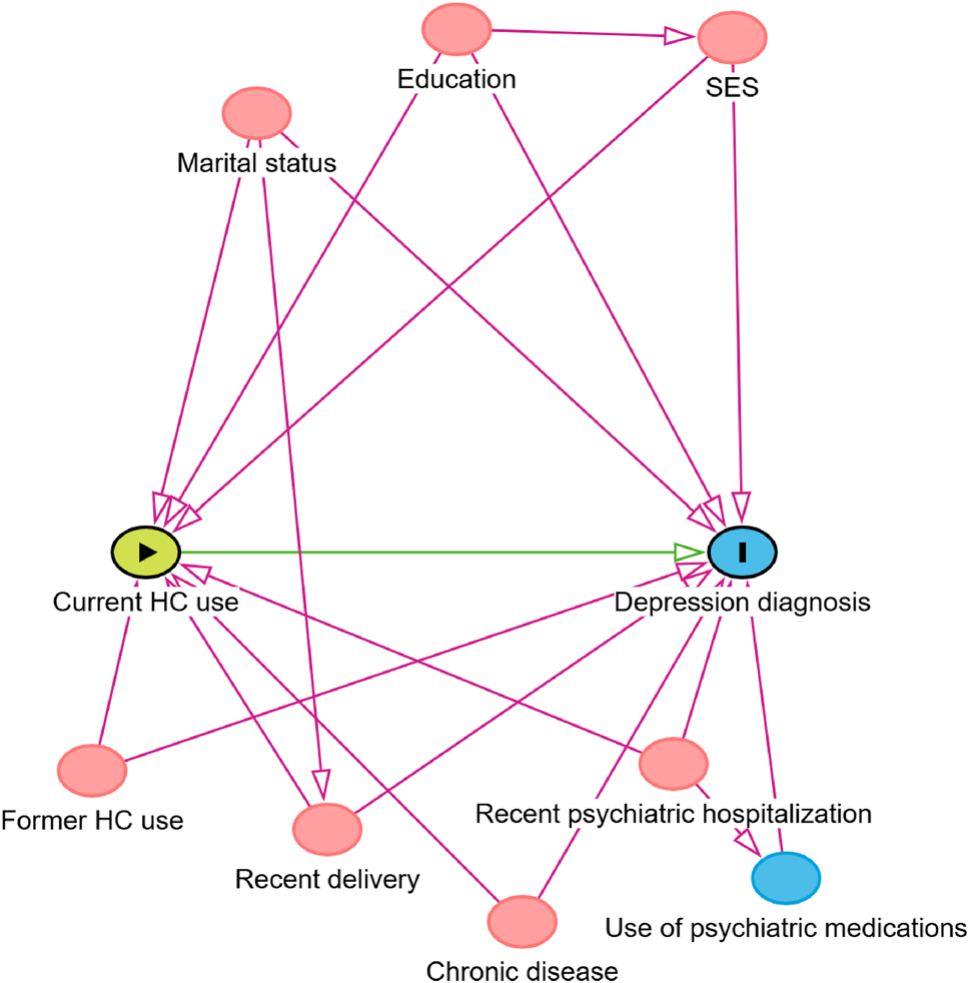
Directed Acyclic Graph (DAG) utilized for model selection

The associations between HC use and depression were examined via conditional logistic regression, which takes matching into account, with group of utilized HC (current use vs. non-use) as the main predictor. In addition to a univariable model, we performed a multivariable Model 2 controlled for marital status, socioeconomic status, education level, recent (in the previous six months or in the previous two years) delivery, and recent (in the previous six months or in the previous two years) psychiatric hospitalizations; and Model 3, further controlled for chronic diseases before start of follow-up, use of psychiatric medications (excluding antidepressants), and former-use of HC (Fig. 1). Because the case and control groups were matched by year of birth, age was not included as covariate in the models; rather, age-stratified analyses were performed. Sensitivity analyses were conducted by only considering depression diagnoses recorded in the Care Register for Health Care (i.e., more severely depressed patients requiring hospitalization or specialized outpatient care). Additionally, to exclude a healthy user bias, adjusted conditional regression analyses were conducted with one redeemed prescription being enough to identify current use of HC.

All the analyses were performed with R software version 4.2.3 [24].

## Results

During the follow-up 1,079,898 person-years were cumulated and 23,480 new-onset depression diagnosis cases observed, with an overall incidence rate (IR) of 21.74 (95% CI = 21.47, 22.02) per 1000 person-years. In the group of HC non-users in 2017, we observed 12,351 new cases of depression (IR = 22.97 per 1000 person-years, 95% CI = 22.57, 23.38), while among HC users there were 11,129 new cases of depression (IR = 20.52, 95% CI = 20.14, 20.91) (Table S2).

For each of the incident 23,480 cases, we selected four age-matched (with 1-year caliber) controls, resulting in a nested case-control study of altogether 117,360 women (Table 1). Compared to the controls, women with a diagnosis of depression were less likely to be married, employed, have tertiary or higher education, and to have given birth in the previous 2 years, but more likely to have a previous psychiatric hospitalization and chronic diseases. Additionally, they were less likely to be current users of HC (18.4% vs. 20.9%), in particular of combined hormonal contraceptives (CHCs) (13.4% vs. 16.3%: ethinylestradiol (EE)-containing preparations, 10.0% vs. 12.1%; estradiol-containing preparations, 3.3% vs. 4.2%; *p* < 0.001). Specifically, current use of all the EE-containing combined preparations (with the exceptions of dienogest and EE, and norelgestromin and EE transdermal patch), of nomegestrol and estradiol, and of cyproterone and estrogen was less common among women with an incident depression diagnosis than in their controls (Table 2).

**Table 1:** Basic characteristics of the nested case-control study of depression

	Care Register for Health Care and Register of Primary Health Care visits			
	Cases (*N* = 23,480)		Controls (*N* = 93,880)	
	N	%	N	%
*Marital status*				
Unmarried	17,664	75.2	70,639	75.2
Married	4285	18.2	19,526	20.8
Divorced	1442	6.1	3487	3.7
Widowed	46	0.2	122	0.1
Other	43	0.2	106	0.1
*Socioeconomic group*				
Self-employed	650	2.8	2934	3.1
Upper-level employees	1561	6.6	9566	10.2
Lower-level employees	5804	24.7	29,295	31.2
Manual workers	3406	14.5	14,385	15.3
Students	6738	28.7	23,046	24.5
Pensioners	500	2.1	1297	1.4
Others	3227	13.7	7687	8.2
Unknown	1594	6.8	5670	6.0
*Education*				
Upper secondary	12,071	51.4	45,454	48.4
Post-secondary non-tertiary	105	0.4	474	0.5
Short-cycle tertiary	286	1.2	1497	1.6
Bachelor	3082	13.1	16,502	17.6
Master	1150	4.9	7787	8.3
Doctoral	42	0.2	374	0.4
Missing (including, e.g., missing information on education other than of primary school level, school dropouts)	6744	28.7	21,792	23.2
*Age group* ^*^				
15–19 years	4296	18.3	17,168	18.3
20–24 years	6795	28.9	27,170	28.9
25–29 years	5125	21.8	20,495	21.8
30–34 years	3083	13.1	12,327	13.1
35–39 years	2052	8.7	8206	8.7
40–44 years	1338	5.7	5350	5.7
45–49 years	791	3.4	3164	3.4
*Previous psychiatric hospitalizations*				
No	13,552	57.7	89,962	95.8
In the previous 6 months	9132	38.9	2080	2.2
6 to 24 months before	796	3.4	1838	2.0
*Cancer in the previous 5 years*	203	0.9	678	0.7
*Chronic diseases at baseline*				
Hypothyroidism	197	0.8	599	0.6
Multiple sclerosis	65	0.3	195	0.2
Epilepsy	328	1.4	866	0.9
Severe psychiatric disorders	532	2.3	774	0.8
Connective tissue diseases	374	1.6	1165	1.2
Ulcerative cholitis or Chron’s disease	237	1.0	735	0.8
Diabetes mellitus	491	2.1	1142	1.2
*Former HC use*	11,129	47.4	47,229	50.3
*Recent delivery*				
No	21,878	93.2	86,601	92.2
In the previous 6 months	315	1.3	1346	1.4
6 to 24 months before	1287	5.5	5933	6.3

^*^ Matched by age

*HC* hormonal contraception

**Table 2:** Hormonal contraception use in the nested case-control study of depression, care register for health care and register of primary health care visits. Cases, *N* = 23,480; controls, *N* = 93,880

	HC use = one redeemed prescription				HC use = two redeemed prescriptions			
	Cases		Controls		Cases		Controls	
	*N*	%	*N*	%	*N*	%	*N*	%
*HC use*								
No HC	16,652	70.9	63,682	67.8	19,150	81.6	74,245	79.1
Current HC	6828	29.1	30,198	32.2	4330	18.4	19,635	20.9
Combined hormonal contraception	4762	20.3	22,866	24.4	3148	13.4	15,264	16.3
Ethinylestradiol containing	3608	15.4	17,505	18.6	2345	10.0	11,364	12.1
Estradiol containing	1154	4.9	5361	5.7	803	3.3	3900	4.2
Progestin-only	2066	8.8	7332	7.8	1182	5.0	4371	4.7
*Combined hormonal contraceptives (ATC code)*								
Levonorgestrel and ethinylestradiol (G03AA07)	91	0.4	400	0.4	49	0.2	243	0.3
Desogestrel and ethinylestradiol (G03AA09)	459	2.0	2329	2.5	264	1.1	1331	1.4
Gestodene and ethinylestradiol (G03AA10)	678	2.9	3622	3.9	431	1.8	2299	2.4
Drospirenone and ethinylestradiol (G03AA12)	1747	7.4	8645	9.2	1082	4.6	5335	5.7
Norelgestromin and ethinylestradiol patch (G03AA13)	105	0.4	297	0.3	75	0.3	212	0.2
Nomegestrol and estradiol (G03AA14)	395	1.7	2165	2.3	228	1.0	1279	1.4
Dienogest and ethinylestradiol (G03AA16)	106	0.5	353	0.4	59	0.3	205	0.2
Dienogest and estradiol-valerate (G03AB08)	186	0.8	836	0.9	124	0.5	486	0.5
Etonogestrel and ethinylestradiol vaginal ring (G02BB01)	484	2.1	2052	2.2	294	1.3	1374	1.5
*Progestin-only oral contraceptives*								
Norethisterone (G03AC01)	184	0.8	618	0.7	104	0.4	355	0.4
Levonorgestrel (G03AC03)	89	0.4	303	0.3	37	0.2	117	0.1
Desogestrel (G03AC09)	1827	7.8	6541	7.0	1022	4.4	3860	4.1
*Antiandrogen and estrogen*								
Cyproterone and estrogen (G03HB01)	630	2.7	2684	2.9	453	1.9	2120	2.3

Consistently, in the univariable logistic regression model, current use of HC (OR = 0.85, 95% CI = 0.82, 0.88), and specifically of CHCs (either EE-containing or estradiol-containing preparations) was associated with lower risk of depression compared to the risk in HC non-users (OR = 0.79, 95% CI = 0.76, 0.82). No significant associations emerged with current use of progestin-only contraceptives (Table 3, bottom). The lower risk associated with CHCs (either EE- or estradiol-containing contraceptives) remained significant after controlling for confounders including former use of HC (Table 3, bottom).

**Table 3:** Associations between current HC use and depression diagnosis (Care register for health care and register of primary health care visits)

		Model 1		Model 2		Model 3	
		Odds Ratio	95% CI	Odds Ratio	95% CI	Odds Ratio	95% CI
*HC use = one redeemed prescription*							
HC use	No HC	Reference		Reference		Reference	
	Current HC	0.86	0.83, 0.89	0.90	0.87, 0.94	0.92	0.87, 0.96
	CHC	0.79	0.76, 0.82	0.86	0.82, 0.89	0.88	0.83, 0.92
	EE containing CHCs	0.78	0.75, 0.81	0.86	0.82, 0.90	0.88	0.83, 0.93
	Estradiol containing CHCs	0.82	0.77, 0.87	0.84	0.78, 0.91	0.85	0.78, 0.93
	Progestin-only	1.08	1.02, 1.13	1.04	0.97, 1.11	1.02	0.95, 1.09
*HC use = two redeemed prescriptions*							
HC use	No HC	Reference		Reference		Reference	
	Current HC	0.85	0.82, 0.88	0.88	0.84, 0.92	0.90	0.85, 0.95
	CHC	0.79	0.76, 0.82	0.84	0.80, 0.88	0.86	0.81, 0.91
	EE containing CHCs	0.79	0.75, 0.83	0.85	0.80, 0.90	0.87	0.82, 0.93
	Estradiol containing CHCs	0.79	0.73, 0.86	0.81	0.74, 0.89	0.82	0.75, 0.91
	Progestin-only	1.04	0.97, 1.11	1.01	0.93, 1.10	1.02	0.93, 1.11

HC use defined as one redeemed prescription (upper part) or two redeemed prescriptions (bottom part). model 1 is univariable; model 2 is controlled for marital status, socioeconomic status, education, recent delivery and recent psychiatric hospitalization; model 3 is model 2 further controlled for chronic diseases*, use of psychiatric medications (excluding antidepressants) and former use of HC

^*^Diabetes, multiple sclerosis, epilepsy, severe psychiatric disorders, connective tissue diseases, ulcerative colitis or Crohn’s disease, cancer

*CHC* combined hormonal contraception, *EE* ethinylestradiol, *HC* hormonal contraception

**Fig. 2:**
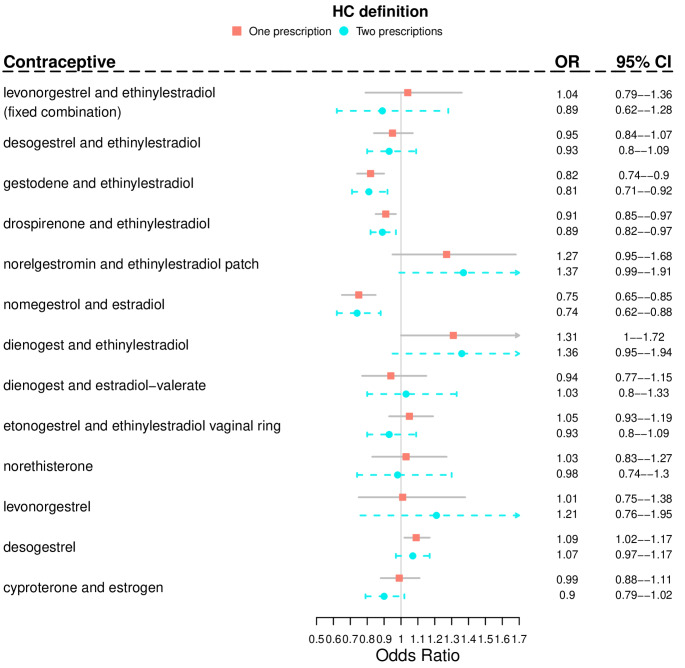
Associations between depression and current use (in the previous 180 days) of hormonal contraceptives. Results are expressed as Odds Ratios with 95% Confidence Intervals. For each substance the reference category is no use of the same substance in the 180 days before the attempted suicide. Adjusted model is controlled for marital status, socioeconomic status, education, recent delivery, recent psychiatric hospitalization, chronic diseases, use of psychiatric medications (excluding antidepressants) and former use of HC. One substance in model a time. Care Register of Health Care and Register of Primary Health Care visits data together

In detail, in the fully adjusted model current use of COCs containing gestodene and EE, drospirenone and EE, and nomegestrol and estradiol was associated with lower risk of depression than non-use of the same preparations (Fig. 2). The results did not substantially change in age-stratified analyses, although the associations did not reach statistical significance in the older age group (35–49 years) (Table 4).

**Table 4:** Age stratified analyses of associations between current HC use and depression diagnosis (Care register for health care and register of primary health care visits)

		15–19 years		20–24 years			25–34 years		35–49 years	
		Odds Ratio	95% CI	Odds Ratio		95% CI	Odds Ratio	95% CI	Odds Ratio	95% CI
		*HC use = one redeemed prescription*								
HC use	No HC	Reference		Reference			Reference		Reference	
	Current HC	0.89	0.79, 1.01	0.86	0.80, 0.92		0.93	0.88, 0.99	0.94	0.86, 1.03
	CHC	0.88	0.78, 1.00	0.82	0.76, 0.89		0.88	0.82, 0.95	0.87	0.77, 0.97
	EE containing CHCs	0.97	0.85, 1.11	0.82	0.76, 0.89		0.87	0.80, 0.94	0.85	0.74, 0.98
	Estradiol containing CHCs	0.64	0.51, 0.81	0.82	0.71, 0.94		0.94	0.82, 1.08	0.90	0.75, 1.09
	Progestin-only	0.96	0.75, 1.21	0.99	0.87, 1.12		1.09	0.98, 1.20	1.04	0.92, 1.18
		*HC use = two redeemed prescriptions*								
	No HC									
	Current HC	0.86	0.75, 0.99	0.83	0.77, 0.90		0.89	0.83, 0.96	0.97	0.87, 1.07
	CHC	0.86	0.74, 0.99	0.81	0.74, 0.88		0.85	0.78, 0.92	0.88	0.77, 1.01
	EE containing CHCs	0.96	0.81, 1.13	0.81	0.74, 0.89		0.85	0.78, 0.94	0.85	0.72, 1.01
	Estradiol containing CHCs	0.65	0.49, 0.85	0.80	0.69, 0.94		0.84	0.72, 0.99	0.93	0.75, 1.15
	Progestin-only	0.86	0.63, 1.19	0.94	0.80, 1.10		1.03	0.90, 1.17	1.09	0.94, 1.26

HC use defined as one redeemed prescription (upper part) or two redeemed prescriptions (bottom part). Analyses are controlled for marital status, socioeconomic status, education, recent delivery and recent psychiatric hospitalization

*CHC* combined hormonal contraception, *EE* ethinylestradiol, *HC* hormonal contraception

Results were unchanged in sensitivity analyses using only one redeemed prescription as indicator of HC use, conducted to exclude a possible “healthy user bias” (Fig. 2; Table 3 upper part, Table 4). The only exceptions were the current use of dienogest and EE, and of desogestrel, which were associated with a higher risk of depression (OR = 1.31, 95% CI = 1.00, 1.72; and OR = 1.09, 95% CI = 1.02, 1.17, respectively) (Fig. 2).

In sensitivity analyses including only the 13,304 depression cases recorded in the Care Register for Health Care and the matched controls (Table S3, Tables S4, S5), the associations between current use of HC and diagnosis of depression lost their statistical significance in the fully adjusted model (Table S6).

## Discussion

Our main finding was that current use of HC was not associated with an increased risk of depression in women of fertile age. Rather, the current use of CHCs, either EE- or estrogen containing preparations, was associated with a lower risk of depression compared to non-use of HC even after controlling for covariates, including former use of HC.

Previous register-based studies from the Nordic countries had reported findings opposite to ours. Our finding that the current use of HC is not associated with an increased risk of depression in fertile-aged women contrasts with results of a large Danish study, finding a higher risk of incident depression (first use of antidepressants and/or first diagnosis) in women aged 15–34 years who were currently or recently (within the previous six months) using either combined (namely, ethinylestradiol in combination with levonorgestrel, desogestrel, gestodene, drospirenone or cyproterone acetate, or natural estrogen in combination with dienogest) or progestin-only (oral norethisterone, levonorgestrel or desogestrel, as well as etonogestrel vaginal ring or norgestrolmin patch) hormonal contraception, when compared to never-users and former users (RR ranging between 1.1 and 2.0) [13]. Similar results were reported by a Swedish study of more than 800,000 women aged 12–30 years, finding higher odds for first use of psychotropic drug in HC users compared to non-users, being more pronounced for non-oral preparations. It is of note that in the Swedish study the association was strong (OR 3.46, 95% CI 3.04–3.94) in adolescent girls, but decreased to non-significance in those older than 20 years of age [14]. It has been found that many women, and up to 82% of teenagers who use OC, do use contraception primarily for reasons other than birth control, e.g., dysmenorrhea, irregular menstrual periods, or acne [25], which are themselves related to depression and anxiety symptoms and disorders [26, 27]. In addition, in the same study the authors found only marginal discriminatory accuracy of HC in identifying psychotropic drug users, suggesting possible residual confounding [14]. Moreover, previous studies found that mental health status, in particular depression in adolescent girls, may influence the choice of contraceptive methods. For example, adolescent girls with depressive symptoms are more likely to choose a long-acting reversible contraceptive (LARC) method than short-acting methods [28], which were not captured in total by our study, but partly captured by earlier studies. It is of note that in our study adolescent girls and young women had the lowest odds for depression in relation to HC use, with an inverse tendency in the older groups. These apparently contradictory findings support the possible role of unmeasured confounding.

It can be argued that our results of lower odds for depression in relation to HC use are in fact explained by a discontinuation bias, where women who develop mood symptoms as side effects of HC discontinue their contraceptive use, thus being categorized as non-users. However, when considering only one redeemed prescription as a definition of HC use (thus including those who possibly stopped using HC due to mood and other side effects), our results did not change. In addition, our adjusted models took a former user category into account. It is of note that, when applying a more stringent definition of depression, i.e., capturing only cases severe enough to receive a diagnosis in a specialized care setting, the associations lost their significance after controlling for a full set of covariates. In line with this observation, it must be acknowledged that the use of ICD and ICPC codes to define our outcome of interest may have caused cases not severe enough to reach a full depression diagnosis to be mistakenly classified as controls. Taken together, and based on these partly opposite findings, our results suggest that use of HC is rather safe in terms of severe mood disorders, although in a subgroup of vulnerable women, possibly those with a pre-existing severe mood condition or belonging to a hormone-sensitive subgroup [11, 12], it may in fact be related to adverse mood symptoms.

This study has some limitations as well. Because this is a register-based study, the definition of HC relied on the redeemed prescriptions rather than on its monitored use in clinical practice. However, because HC is not reimbursable by the Social Insurance Institution of Finland, it is likely that most women who had purchased the drug did use it as prescribed. Additionally, we lacked information on the precise contents of the contraceptive preparations used, which precluded any analyses on the effect of different doses of EE. Similarly, information regarding non-hormonal methods (e.g., copper intrauterine device, barrier methods, etc.) as well as contraceptives obtained free-of-charge as part of municipal programs, especially those for LARC methods, was not available. Furthermore, we cannot exclude that the associations we found are confounded by some external unaccounted factors, such as a relationship status variable accounting, for example, for those who were in a stable relationship but unmarried. Additionally, although we control part of the analyses for former HC use, results may still have been impacted by residual confounding related to former HC use.

Among the strengths of our study is the use of Finnish register data of proven high quality [29], and the identification of depression cases based on diagnostic codes. The nested case-control design produces unbiased estimates and is free from weaknesses of the ordinary case-control design. It uses correct sampling of controls that takes the follow-up time into account [30, 31]. In addition, the control women were matched by age. Thus, our study design provides results that are relatively free from confounding bias, although some residual confounding is always possible in observational studies.

Taken together, our results convey the reassuring message to fertile-aged women seeking contraception that HC use is not associated with an increased risk of severe depressive disorder. At the same time, they stress the importance of considering personalized choice of the best and safest contraceptive option for each woman.

## Electronic supplementary material

Below is the link to the electronic supplementary material.

Supplementary Material 1

Supplementary Material 2

Supplementary Material 3

Supplementary Material 4

Supplementary Material 5

Supplementary Material 6

## Data Availability

The data that support the findings of this study are available from Statistics Finland, the Finnish Institute for Health and Welfare, and the Social Insurance Institution, but restrictions apply to the availability of these data, which were used under license for the current study, and so are not publicly available. Data are, however, available from the authors upon reasonable request and with permission of FinData (https://www.findata.fi/en/).
