# Supplementary material for: Use of systemic hormonal contraception and risk of depression: a registry-based study from Finland
Source: Eur J Epidemiol. 2025 Jul 2;40(8):915–23. doi: 10.1007/s10654-025-01267-0 (PMC12374907; doi:10.1007/s10654-025-01267-0)
Supplement: Supplementary file 1 — Supplementary Material 1 [file 10654_2025_1267_MOESM1_ESM.docx]

**Table S1. Systemic hormonal contraceptives, the doses available and their use in Finland in 2018 (https://www.fimea.fi/web/en/databases_and_registers/fimeaweb).**

| **ATC code** | **Available doses** | | **Use in 2018^*^** |
| --- | --- | --- | --- |
| **Combined hormonal contraceptives** | **Monophasic** | **Phasic** |  |
| G03AA07 (levonorgestrel and ethinylestradiol) | 150 μg and 30 μg | 50-125 μg and 30-40 μg | 0.68 |
| G03AA09 (desogestrel and ethinylestradiol) | 150 μg and 20/30 μg | 25-125 μg and 30-40 μg | 3.44 |
| G03AA10 (gestodene and ethinylestradiol) | 75 μg and 20/30 μg | 20-100 μg and 30-40 μg | 5.13 |
| G03AA11 (norgestimate and ethinylestradiol) | 250 μg and 35 μg |  | 0.04 |
| G03AA12 (drospirenone and ethinylestradiol) | 3 mg and 20/30 μg |  | 11.3 |
| G03AA13 (norelgestromin and ethinylestradiol patch) | 6 mg and 600 μg |  | 0.48 |
| G03AA14 (nomegestrol and estradiol) | 2.5 mg and 1.5 mg |  | 3.13 |
| G03AA16 (dienogest and ethinylestradiol) | 2 mg and 30 μg |  | 0.47 |
| G03AB08 (dienogest and estradiol-valerate) |  | 2-3 mg and 1-3 mg | 1.36 |
| G02BB01 (etonogestrel and ethinylestradiol vaginal ring) | 120 μg and 15 μg |  | 2.86 |
| **Progestin-only oral contraceptives** |  |  |  |
| G03AC01 (norethisterone) | 350 μg |  | 0.12 |
| G03AC03 (levonorgestrel) | 30 μg |  | 0.45 |
| G03AC09 (desogestrel) | 75 μg |  | 10.93 |
| **Antiandrogen and estrogen** |  |  |  |
| G03HB01 (cyproterone and estrogen) | 2 mg and 35 μg (EE) | 1-2 mg and 1-2 mg (E2) | 3.34 |

^*^ Defined daily doses (DDD) / 1000 inhabitants/day
