# Supplementary material for: Use of systemic hormonal contraception and risk of depression: a registry-based study from Finland
Source: Eur J Epidemiol. 2025 Jul 2;40(8):915–23. doi: 10.1007/s10654-025-01267-0 (PMC12374907; doi:10.1007/s10654-025-01267-0)
Supplement: Supplementary file 2 — Supplementary Material 2 [file 10654_2025_1267_MOESM2_ESM.docx]

**Table S2. Incidence of depression. Cases based on diagnosis (Care Register for Health Care and Register of Primary Health Care visits).** Incidence rate ratio (IRR) with 95% confidence interval based on Poisson regression model.

|  |  | **Person-years** | **Events** | **Rate (1/1000)** | **95% CI** | **IRR** | **95% CI** |
| --- | --- | --- | --- | --- | --- | --- | --- |
| **HC use in year 2017** | no HC | 537,601 | 12,351 | 22.97 | 22.57, 23.38 | reference | reference |
|  | HC | 542,297 | 11,129 | 20.52 | 20.14, 20.91 | 0.89 | 0.87, 0.92 |
| **Age group** | 15-19 years | 137,315 | 4296 | 31.29 | 30.36, 32.24 | reference | reference |
|  | 20-24 years | 253,752 | 6795 | 26.78 | 26.15, 27.42 | 0.86 | 0.82, 0.89 |
|  | 25-29 years | 233,900 | 5125 | 21.91 | 21.32, 22.52 | 0.70 | 0.67, 0.73 |
|  | 30-34 years | 164,203 | 3083 | 18.78 | 18.12, 19.45 | 0.60 | 0.57, 0.63 |
|  | 35-39 years | 126,089 | 2052 | 16.27 | 15.58, 16.99 | 0.52 | 0.49, 0.55 |
|  | 40-44 years | 95,258 | 1338 | 14.05 | 13.30, 14.82 | 0.45 | 0.42, 0.48 |
|  | 45-49 years | 69,382 | 791 | 11.40 | 10.62, 12.22 | 0.36 | 0.34, 0.39 |
| **Socioeconomic group** | Self-employed | 39,334 | 650 | 16.53 | 15.28, 17.85 | reference | reference |
|  | Upper-level employees | 138,283 | 1561 | 11.29 | 10.74, 11.86 | 0.33 | 0.18, 0.62 |
|  | Lower-level employees | 360,522 | 5804 | 16.10 | 15.69, 16.52 | 0.68 | 0.42, 1.10 |
|  | Manual workers | 162,244 | 3406 | 20.99 | 20.29, 21.71 | 1.40 | 0.86, 2.28 |
|  | Students | 210,887 | 6738 | 31.95 | 31.19, 32.72 | 2.56 | 1.61, 4.07 |
|  | Pensioners | 16,170 | 500 | 30.92 | 28.27, 33.75 | 7.45 | 4.49, 12.37 |
|  | Others | 88,491 | 3227 | 36.47 | 35.22, 37.75 | 2.62 | 1.62, 4.25 |
|  | Unknown | 63,968 | 1594 | 24.92 | 23.71, 26.17 | 2.48 | 1.49, 4.11 |
| **Education** | Upper secondary | 496,732 | 12,071 | 24.30 | 23.87, 24.74 | reference | reference |
|  | Post-secondary non-tertiary | 7,157 | 105 | 14.67 | 12.00, 17.76 | 0.60 | 0.50, 0.73 |
|  | Short-cycle tertiary | 29,516 | 286 | 9.69 | 8.60, 10.88 | 0.40 | 0.36, 0.45 |
|  | Bachelor | 212,428 | 3082 | 14.51 | 14.00, 15.03 | 0.60 | 0.57, 0.62 |
|  | Master | 119,806 | 1150 | 9.60 | 9.05, 10.17 | 0.40 | 0.37, 0.42 |
|  | Doctoral | 6,393 | 42 | 6.57 | 4.74, 8.88 | 0.27 | 0.20, 0.37 |
|  | Unknown | 207,867 | 6744 | 32.44 | 31.67, 33.23 | 1.34 | 1.30, 1.38 |
| **Marital status** | Unmarried | 733,164 | 17,664 | 24.09 | 23.74, 24.45 | reference | reference |
|  | Married | 286,751 | 4285 | 14.94 | 14.50, 15.40 | 0.62 | 0.60, 0.64 |
|  | Divorced | 56,695 | 1442 | 25.43 | 24.14, 26.78 | 1.06 | 1.00 1.11 |
|  | Widowed | 1935 | 46 | 23.77 | 17.40, 31.71 | 0.99 | 0.74, 1.32 |
|  | Other | 1354 | 43 | 31.76 | 22.99, 42.79 | 0.78 | 0.98, 1.78 |

HC, hormonal contraception
