# Supplementary material for: Use of systemic hormonal contraception and risk of depression: a registry-based study from Finland
Source: Eur J Epidemiol. 2025 Jul 2;40(8):915–23. doi: 10.1007/s10654-025-01267-0 (PMC12374907; doi:10.1007/s10654-025-01267-0)
Supplement: Supplementary file 3 — Supplementary Material 3 [file 10654_2025_1267_MOESM3_ESM.docx]

**Table S3. Incidence of depression,** only cases from Care Register for Health Care included**.** Incidence rate ratio (IRR) with 95% confidence interval based on Poisson regression model.

|  |  | **Person-years** | **Events** | **Rate (1/1000)** | **95% CI** | **IRR** | **95% CI** |
| --- | --- | --- | --- | --- | --- | --- | --- |
| **HC use in year 2017** | no HC | 555,727 | 7075 | 12.73 | 12.44, 13.03 | reference | reference |
|  | HC | 559,593 | 6229 | 11.13 | 10.86, 11.41 | 0.87 | 0.85, 0.91 |
| **Age group** | 15-19 years | 141,795 | 2687 | 18.95 | 18.24, 19.68 | reference | reference |
|  | 20-24 years | 264,489 | 3740 | 14.14 | 13.69, 14.60 | 0.75 | 0.71, 0.78 |
|  | 25-29 years | 242,392 | 2896 | 11.95 | 11.52, 12.39 | 0.63 | 0.60, 0.66 |
|  | 30-34 years | 169,171 | 1660 | 9.81 | 9.35, 10.30 | 0.52 | 0.49, 0.55 |
|  | 35-39 years | 129,322 | 1145 | 8.85 | 8.35, 9.38 | 0.47 | 0.44, 0.50 |
|  | 40-44 years | 97,413 | 718 | 7.37 | 6.84, 7.93 | 0.39 | 0.36, 0.42 |
|  | 45-49 years | 70,739 | 458 | 6.47 | 5.90, 7.10 | 0.34 | 0.31, 0.38 |
| **Socioeconomic group** | Self-employed | 40,315 | 319 | 7.91 | 7.07, 8.83 | reference | reference |
|  | Upper-level employees | 140,521 | 881 | 6.27 | 5.86, 6.70 | 0.79 | 0.70, 0.90 |
|  | Lower-level employees | 368,970 | 3174 | 8.60 | 8.31, 8.91 | 1.09 | 0.97, 1.22 |
|  | Manual workers | 167,161 | 1815 | 10.86 | 10.36, 11.37 | 1.37 | 1.22, 1.55 |
|  | Students | 221,348 | 3974 | 17.95 | 17.40, 18.52 | 2.27 | 2.02, 2.54 |
|  | Pensioners | 17,098 | 329 | 19.24 | 17.22, 21.44 | 2.43 | 2.09, 2.84 |
|  | Others | 93,458 | 1825 | 19.53 | 18.64, 20.44 | 2.47 | 2.19, 2.78 |
|  | Unknown | 66,449 | 987 | 14.85 | 13.94, 15.81 | 1.88 | 1.66, 2.13 |
| **Education** | Upper secondary | 515,686 | 6551 | 12.70 | 12.40, 13.02 | reference | reference |
|  | Post-secondary non-tertiary | 7286 | 50 | 6.86 | 5.09, 9.05 | 0.54 | 0.41, 0.71 |
|  | Short-cycle tertiary | 30,016 | 160 | 5.33 | 4.54, 6.22 | 0.42 | 0.36, 0.49 |
|  | Bachelor | 217,870 | 1645 | 7.55 | 7.19, 7.92 | 0.59 | 0.56, 0.63 |
|  | Master | 121,579 | 672 | 5.53 | 5.12, 5.96 | 0.44 | 0.40, 0.47 |
|  | Doctoral | 6433 | 27 | 4.20 | 2.77, 6.11 | 0.33 | 0.23, 0.48 |
|  | Unknown | 216,450 | 4199 | 19.40 | 18.82, 20.00 | 1.53 | 1.47, 1.59 |
| **Marital status** | Unmarried | 759,470 | 10,179 | 13.40 | 13.14, 13.67 | reference | reference |
|  | Married | 293,180 | 2293 | 7.82 | 7.50, 8.15 | 0.58 | 0.56, 0.61 |
|  | Divorced | 59,202 | 787 | 13.29 | 12.38, 14.26 | 0.99 | 0.92, 1.07 |
|  | Widowed | 2039 | 20 | 9.81 | 5.99, 15.15 | 0.73 | 0.47, 1.14 |
|  | Other | 1429 | 25 | 17.50 | 11.32, 25.83 | 1.31 | 0.88, 1.93 |

HC, hormonal contraception; IRR, Incidence Rate Ratio
