# Supplementary material for: Use of systemic hormonal contraception and risk of depression: a registry-based study from Finland
Source: Eur J Epidemiol. 2025 Jul 2;40(8):915–23. doi: 10.1007/s10654-025-01267-0 (PMC12374907; doi:10.1007/s10654-025-01267-0)
Supplement: Supplementary file 4 — Supplementary Material 4 [file 10654_2025_1267_MOESM4_ESM.docx]

**Table S4. Basic characteristics of the nested case-control study of depression,** Cases based on diagnosis from Care Register for Health Care only.

|  | **Cases**  **(N=13,303)** | | **Controls**  **(N=53,204)** | |  |
| --- | --- | --- | --- | --- | --- |
|  | **N** | **%** | **N** | **%** | **p-value** |
| **Marital status** |  |  |  |  | <0.001 |
| Unmarried | 10,178 | 76.5 | 40,348 | 75.8 |  |
| Married | 2293 | 17.2 | 10,697 | 20.1 |  |
| Divorced | 787 | 5.9 | 2036 | 3.8 |  |
| Widowed | 20 | 0.2 | 72 | 0.1 |  |
| Other | 25 | 0.2 | 51 | 0.1 |  |
| **Socioeconomic group** |  |  |  |  | <0.001 |
| Self-employed | 319 | 2.4 | 1655 | 3.1 |  |
| Upper-level employees | 881 | 6.6 | 5690 | 10.7 |  |
| Lower-level employees | 3174 | 23.9 | 16,107 | 30.3 |  |
| Manual workers | 1815 | 13.6 | 7995 | 15.0 |  |
| Students | 3973 | 29.9 | 13,590 | 25.5 |  |
| Pensioners | 329 | 2.5 | 741 | 1.4 |  |
| Others | 1825 | 13.7 | 4203 | 7.9 |  |
| Unknown | 987 | 7.4 | 3223 | 6.1 |  |
| **Education** |  |  |  |  | <0.001 |
| Upper secondary | 6551 | 49.2 | 24,825 | 46.7 |  |
| Post-secondary non-tertiary | 50 | 0.4 | 248 | 0.5 |  |
| Short-cycle tertiary | 160 | 1.2 | 815 | 1.5 |  |
| Bachelor | 1645 | 12.4 | 9052 | 17.0 |  |
| Master | 672 | 5.1 | 4560 | 8.6 |  |
| Doctoral | 27 | 0.2 | 225 | 0.4 |  |
| Missing (including, e.g., missing information on  education other than of primary school level,  school dropouts) | 4198 | 31.6 | 13,479 | 25.3 |  |
| **Age group** |  |  |  |  | 1.000 |
| 15-19 years | 2686 | 20.2 | 10,741 | 20.2 |  |
| 20-24 years | 3740 | 28.1 | 14,957 | 28.1 |  |
| 25-29 years | 2896 | 21.8 | 11,583 | 21.8 |  |
| 30-34 years | 1660 | 12.5 | 6639 | 12.5 |  |
| 35-39 years | 1145 | 8.6 | 4580 | 8.6 |  |
| 40-44 years | 718 | 5.4 | 2872 | 5.4 |  |
| 45-49 years | 458 | 3.4 | 1832 | 3.4 |  |
| **Previous psychiatric hospitalizations** |  |  |  |  | <0.001 |
| No | 300 | 2.3 | 50,664 | 95.2 |  |
| In the previous 6 months | 12,979 | 97.6 | 1486 | 2.8 |  |
| 6 to 24 months before | 24 | 0.2 | 1054 | 2.0 |  |
| **Cancer in the previous 5 years** | 117 | 0.9 | 419 | 0.8 | 0.314 |
| **Chronic diseases at baseline** |  |  |  |  |  |
| Hypothyroidism | 112 | 0.8 | 419 | 0.8 | 0.003 |
| Multiple sclerosis | 39 | 0.3 | 103 | 0.2 | 0.034 |
| Epilepsy | 215 | 1.6 | 489 | 0.9 | <0.001 |
| Severe psychiatric disorders | 387 | 2.9 | 461 | 0.9 | <0.001 |
| Connective tissue diseases | 232 | 1.7 | 586 | 1.1 | <0.001 |
| Ulcerative cholitis or Chron’s disease | 152 | 1.1 | 452 | 0.8 | 0.002 |
| Diabetes mellitus | 317 | 2.4 | 633 | 1.2 | <0.001 |
| **Former HC use** | 6229 | 46.8 | 26,715 | 50.2 | <0.001 |
| **Recent delivery** |  |  |  |  | <0.001 |
| No | 12,579 | 94.6 | 49,405 | 92.9 |  |
| In the previous 6 months | 134 | 1.0 | 656 | 1.2 |  |
| 6 to 24 months before | 590 | 4.4 | 3143 | 5.9 |  |

HC, hormonal contraception.
