# Supplementary material for: Use of systemic hormonal contraception and risk of depression: a registry-based study from Finland
Source: Eur J Epidemiol. 2025 Jul 2;40(8):915–23. doi: 10.1007/s10654-025-01267-0 (PMC12374907; doi:10.1007/s10654-025-01267-0)
Supplement: Supplementary file 5 — Supplementary Material 5 [file 10654_2025_1267_MOESM5_ESM.docx]

**Table S5. Hormonal contraception use in the nested case-control study of depression,** Cases based on diagnosis from Care Register for Health Care only. Cases, N=13,303; Controls, N = 53,204

|  | **HC use = one redeemed prescription** | | | | **HC use = two redeemed prescriptions** | | | |
| --- | --- | --- | --- | --- | --- | --- | --- | --- |
|  | **Cases** | | **Controls** | | **Cases** | | **Controls** | |
|  | **N** | **%** | **N** | **%** | **N** | **%** | **N** | **%** |
| **HC use** |  |  |  |  |  | |  | |
| No HC | 9681 | 72.8 | 36,677 | 68.9 | 11,030 | 82.9 | 42,690 | 80.2 |
| Current HC | 3622 | 27.2 | 16,527 | 31.1 | 2273 | 17.1 | 10,514 | 19.8 |
| Combined hormonal contraception | 2508 | 18.9 | 12,469 | 23.4 | 1653 | 12.4 | 8117 | 15.3 |
| Ethinylestradiol containing | 1875 | 14.1 | 9602 | 18.0 | 1218 | 9.2 | 6033 | 11.3 |
| Estradiol containing | 633 | 4.8 | 2867 | 5.4 | 435 | 3.3 | 2084 | 3.9 |
| Progestin-only | 1114 | 8.4 | 4058 | 7.6 | 620 | 4.7 | 2397 | 4.5 |
| **Combined hormonal contraceptives (ATC code)** |  |  |  |  |  |  |  | |
| Levonorgestrel and ethinylestradiol (G03AA07) | 91 | 0.4 | 400 | 0.4 | 21 | 0.2 | 139 | 0.3 |
| Desogestrel and ethinylestradiol (G03AA09) | 459 | 2.0 | 2329 | 2.5 | 117 | 0.9 | 677 | 1.3 |
| Gestodene and ethinylestradiol (G03AA10) | 678 | 2.9 | 3622 | 3.9 | 212 | 1.6 | 1208 | 2.3 |
| Drospirenone and ethinylestradiol (G03AA12) | 1747 | 7.4 | 8645 | 9.2 | 593 | 4.5 | 2796 | 5.3 |
| Norelgestromin and ethinylestradiol patch (G03AA13) | 105 | 0.4 | 297 | 0.3 | 38 | 0.3 | 137 | 0.3 |
| Nomegestrol and estradiol (G03AA14) | 395 | 1.7 | 2165 | 2.3 | 112 | 0.8 | 577 | 1.1 |
| Dienogest and ethinylestradiol (G03AA16) | 106 | 0.5 | 353 | 0.4 | 37 | 0.3 | 148 | 0.3 |
| Dienogest and estradiol-valerate (G03AB08) | 186 | 0.8 | 836 | 0.9 | 70 | 0.5 | 284 | 0.5 |
| Etonogestrel and ethinylestradiol vaginal ring (G02BB01) | 484 | 2.1 | 2052 | 2.2 | 152 | 1.1 | 747 | 1.4 |
| **Progestin-only oral contraceptives** |  |  |  |  |  | |  | |
| Norethisterone (G03AC01) | 184 | 0.8 | 618 | 0.7 | 49 | 0.4 | 198 | 0.4 |
| Levonorgestrel (G03AC03) | 89 | 0.4 | 303 | 0.3 | 19 | 0.1 | 45 | 0.1 |
| Desogestrel (G03AC09) | 1827 | 7.8 | 6541 | 7.0 | 542 | 4.1 | 2130 | 4.0 |
| **Antiandrogen and estrogen** |  |  |  |  |  | |  | |
| Cyproterone and estrogen (G03HB01) | 630 | 2.7 | 2684 | 2.9 | 258 | 1.9 | 1223 | 2.3 |

HC, hormonal contraception.
