# Supplementary material for: Use of systemic hormonal contraception and risk of depression: a registry-based study from Finland
Source: Eur J Epidemiol. 2025 Jul 2;40(8):915–23. doi: 10.1007/s10654-025-01267-0 (PMC12374907; doi:10.1007/s10654-025-01267-0)
Supplement: Supplementary file 6 — Supplementary Material 6 [file 10654_2025_1267_MOESM6_ESM.docx]

**Table S6. Associations between current HC use and diagnosis of depression,** Cases based on diagnosis (Care Register for Health Care only). Model 1 is univariable; Model 2 is controlled for marital status, socioeconomic status, education, recent delivery and recent psychiatric hospitalization; Model 3 is Model 2 further controlled for chronic diseases*, use of psychiatric medications (excluding antidepressants) and former use of HC.

|  | | **Model 1** | | **Model 2** | | **Model 3** | |
| --- | --- | --- | --- | --- | --- | --- | --- |
|  |  | **Odds Ratio** | **95% CI** | **Odds Ratio** | **95% CI** | **Odds Ratio** | **95% CI** |
| **HC use = one redeemed prescription** | | | | | | | |
| **HC use** | No HC | reference | | reference | | reference | |
|  | Current HC | *0.82* | *0.79, 0.86* | *0.77* | *0.66, 0.88* | 1.04 | 0.87, 1.23 |
|  | CHC | *0.75* | *0.72, 0.79* | *0.75* | *0.64, 0.89* | 1.05 | 0.87, 1.27 |
|  | EE containing CHCs | *0.73* | *0.69, 0.77* | *0.75* | *0.63, 0.90* | 1.05 | 0.85, 1.28 |
|  | Estradiol containing CHCs | *0.83* | *0.76, 0.91* | *0.75* | *0.56, 1.02* | 1.08 | 0.78, 1.50 |
|  | Progestin-only | 1.04 | 0.97, 1.11 | 0.79 | 0.63, 1.01 | 1.00 | 0.77, 1.30 |
| **HC use = two redeemed prescriptions** | | | | | | | |
| **HC use** | No HC | reference | | reference | | reference | |
|  | Current HC | *0.83* | *0.79, 0.87* | *0.78* | *0.65, 0.92* | 1.03 | 0.85, 1.25 |
|  | CHC | *0.78* | *0.73, 0.82* | *0.77* | *0.64, 0.94* | 1.05 | 0.84, 1.30 |
|  | EE containing CHCs | *0.77* | *0.72, 0.82* | 0.83 | 0.67, 1.03 | 1.12 | 0.88, 1.42 |
|  | Estradiol containing CHCs | *0.80* | *0.72, 0.89* | *0.63* | *0.44, 0.91* | 0.85 | 0.58, 1.26 |
|  | Progestin-only | 0.99 | 0.91, 1.09 | 0.78 | 0.57, 1.07 | 0.96 | 0.69, 1.35 |

^*^diabetes, multiple sclerosis, epilepsy, severe psychiatric disorders, connective tissue diseases, ulcerative colitis or Crohn’s disease, cancer

CHC, Combined hormonal contraception; EE, ethinylestradiol; HC, hormonal contraception
